# Favorable Effects of Atrial Fixation Transcatheter Mitral Valve Replacement on Left Atrium Volume and Strain

**DOI:** 10.1016/j.jacadv.2025.102236

**Published:** 2025-10-16

**Authors:** Nadira Hamid, Paul Grayburn, Vlasis Ninios, Krzysztof Wrobel, Naeem Tahirkheli, Ron Waksman, Michael Rinaldi, Marek Grygier, Philippe Genereux, Paul Sorajja

**Affiliations:** a Minneapolis Heart Institute, Minneapolis, Minnesota, USA; b Baylor Scott & White The Heart Hospital, Plano, Texas, USA; c Interbalkan Medical Center, Thessaloniki, Greece; d Warsaw Medicover Hospital, Warsaw, Poland; e Lazarski University, Warsaw, Poland; f Oklahoma Heart Hospital, Oklahoma City, Oklahoma; g MedStar Washington Hospital Center, Washington, DC, USA; h Atrium Health Sanger Heart and Vascular Institute, Charlotte, North Carolina, USA; i 1^st^ Department of Cardiology, Poznan University of Medical Sciences, Poznan, Poland; j Gagnon Cardiovascular Institute at Morristown Medical Center, Morristown, New Jersey, USA

**Keywords:** left atrial volume, left atrial strain, mitral regurgitation, transcatheter mitral valve replacement

## Abstract

**Background:**

The AltaValve System transcatheter mitral valve replacement (TMVR) has a spherical nitinol frame designed to fit the left atrium (LA) in a compliant fashion.

**Objectives:**

The objective of the study was to evaluate the anatomical and functional changes of the LA with the AltaValve System.

**Methods:**

A total of 16 patients with severe, symptomatic mitral regurgitation who underwent AltaValve System transcatheter mitral valve replacement implantation via either a transseptal (n = 8) or transapical (n = 8) approach were examined. Transthoracic echocardiography was performed at baseline and 6-month follow-up. LA volumes and strain were evaluated using dedicated software (TomTec) with paired statistical testing.

**Results:**

All patients (median age, 77 years; 75% women [12/16]) had successful implantation. Most had atrial fibrillation (87%) and normal/reduced left ventricular ejection fraction. There was significant reduction in LA volume at 6 months (117.4 ± 39 mL vs 93.0 ± 37.0 mL; *P* = 0.002). For LA strain, there was significant reduction in both reservoir (10.8% ± 5.8% vs 1.6% ± 2.1%; *P* < 0.001) and conduit phase (−9.7% ± 3.2% vs −4.2% ± 4.4%; *P* = 0.013) of LA cycle. There were no significant change in the contraction phase (−1.1% vs −2.7%; *P* = 0.079). There was significant improvement in exercise capacity among 13 patients (average 6-minute walk test distance from 253 m at baseline to 296 m) and in functional status 55.1 ± 20.5 at baseline to 67.0 ± 22.5 (*P* = 0.04) at 6-month follow-up. For the responders, there were statistically significant differences at 6-month follow-up for majority of the LA parameters including strain.

**Conclusions:**

There was a presence of LA remodeling following AltaValve System implantation and this was accompanied by a reduction in LA strain for both the reservoir and conduit phases, without changes in contractility.

Mitral regurgitation (MR) is the most common valvular heart disease and is associated with increased morbidity and mortality when left untreated.^1^ The AltaValve System (4C Medical Technologies, Inc) is novel transcatheter mitral valve replacement (TMVR) technology designed to address anatomical limitations. The AltaValve System implant consists of: 1) self-expanding nitinol frame (cage) that is secured within left atrium (LA) by oversizing to the LA width and height and marginally to the mitral valve annulus for sealing; and 2) prosthetic valve that is secured via the inner frame above the native mitral valve. The implant has minimal left ventricle footprint, and deformation of the outer frame is isolated and do not affect prosthetic valve function, that was purposefully designed for AltaValve System to expand the range of treatable MR patients.

LA remodeling effects have been observed in other valvular repair and replacement technologies. In this study, we aimed to evaluate the anatomical and functional changes of the LA from the baseline to 6 months after treatment with AltaValve System.

## Methods

**Figure 1:**
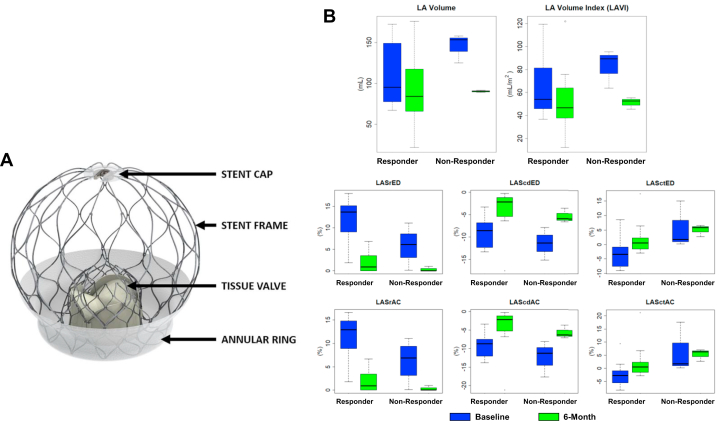
**AltaValve TMVR LA Remodeling and Strain in Responders vs Nonresponders** (A) Overview of AltaValve transcatheter mitral valve replacement. (B) LA Vol., LA volume index (LAVI), LA reservoir strain end diastole (LASrED), LA conduit strain at end diastole (LAScdED), LA contraction strain at end diastole (LASctED), LA reservoir strain at atrial contraction (LASrAC), LA conduit strain at atrial contraction (LAScdAC), and LA contraction strain at atrial contraction (LASctAC) at baseline (blue) and 6-month (green) in responders and nonresponders. LA = left atrium; TMVR = transcatheter mitral valve replacement.

This retrospective analysis of echocardiographic imaging assessed the impact of the AltaValve TMVR on the left atrial volume and strain in EFS (AltaValve Early Feasibility Study) subjects. The Institutional Review Board of all participating sites approved the trial, and all patients provided written informed consent. AltaValve EFS participants were treated using either the transapical or transseptal approach. The AltaValve System implant’s cage is sized individually for each patient with the ventricular end, termed the annular ring, covered by a fabric skirt to minimize the paravalvular leak (Figure 1A). The annular ring is available in 3 sizes (40 mm, 46 mm, and 54 mm) to accommodate variations in native mitral valve anatomy and LA shapes and volumes.

Transthoracic echocardiography was performed using a standardized protocol at each participating site. Speckle tracking echocardiography analysis was performed with an offline workstation (TomTec Imaging Systems LA Strain Analysis) using 4-chamber and 2-chamber cine images. Changes in echocardiographic parameters at baseline and 6-month follow-up were compared, including transthoracic echocardiography, 6-minute walk test (6MWT), Kansas City Cardiomyopathy Questionnaire (KCCQ) overall summary score and NYHA functional class. These were performed as part of a nonrandomized, nonblinded cohort. A responder analysis was performed using KCCQ score. Responders were defined as subjects who reported an increase of ≥10 points at their 6-month follow-up as compared to baseline.

Continuous variables were presented as the mean ± SD (n), [range] and were compared using the paired samples *t*-test or the Wilcoxon signed-rank test, when normally or non-normally distributed, respectively. Normality for continuous variables was assessed using the Shapiro-Wilk test. Categoric variables were summarized as frequencies (proportions). A 2-sided value of *P* < 0.05 was considered significant. Statistical analyses were performed using R statistical software (version 4.4.0).

## Results

Sixteen consecutive subjects with severe MR who were treated with AltaValve System in the AltaValve EFS were analyzed retrospectively. Degenerative or mixed MR etiologies, classified as primary MR, were identified in 31.3% (5/16) of subjects. Functional MR was present in the remaining 68.8% (11/16). Echocardiographic parameters demonstrated preserved or low normal left ventricular ejection fraction (54.6 ± 8.1) and a mean LA volume of 117.4 ± 38.5 mL. All 16 subjects (100.0%) underwent successful implantation of the AltaValve System device; 50.0% (8/16) transapical and 50.0% (8/16) transseptal access. No intraprocedural mortality, device malposition, or embolization events were observed. Discharge echocardiographic assessment revealed successful reduction of MR severity in all subjects to none or trace paravalvular leak.

At 6-month follow-up, 15 subjects had analyzable echocardiographic images. Sustained MR reduction was achieved in all subjects (80% none/trace, 20% mild). Paired analyses showed favorable LA remodeling, with reductions in LA diameter (−0.5 ± 0.6 cm; *P* < 0.01) and left atrium volume index (−13.6 ± 14.5 mL/m^2^; *P* < 0.01). Speckle-tracking revealed significant declines in LA reservoir strain (10.8% ± 5.8% to 1.6% ± 2.1%; *P* < 0.001) and conduit strain (−9.7% ± 3.2% to −4.2% ± 4.4%; *P* = 0.01), with no significant change in contractile strain −1.1% vs 2.7%; *P* = 0.08) (Figure 1B). Improvements in the LA size correlated with sustained clinical benefit, as reflected in NYHA functional class improvement at 6 months despite reduced strain measures.

6MWT results showed improved exercise capacity in 13 subjects. Three did not complete the test due to noncardiac physical limitations. The mean 6MWT distance increased from 253 m at baseline to 296 m at 6 months. Follow-up KCCQ data indicated significant functional improvement, with 15 subjects showing a mean score increase from 55.1 ± 20.5 to 67.0 ± 22.5 (*P* = 0.04). One subject was excluded due to an unmeasurable baseline score.

Among 15 with valid baseline and 6-month KCCQ scores, 73% (11/15) were responders and 27% (4/15) nonresponders. The mean KCCQ change was +21.3 ± 12.1 in responders and −13.9 ± 14.4 in nonresponders. Responders showed significant improvements in most LA parameters, including strain. Nonresponders had no significant changes in LA strain despite a significant reduction in LA volume (*P* = 0.029).

## Discussion

This first echocardiographic study of LA mechanics post-AltaValve System TMVR implantation in symptomatic severe MR patients found: 1) significant reductions in LA diameter and volume at 6-month follow-up; 2) decreased reservoir and conduit strain in responders, with unchanged contractility; 3) no significant LA strain changes in nonresponders despite volume reduction; and 4) elimination of MR and presence of LA remodeling post-AltaValve TMVR implantation correlated with improved quality of life and functional outcomes (Central Illustration).

**Central Illustration:**
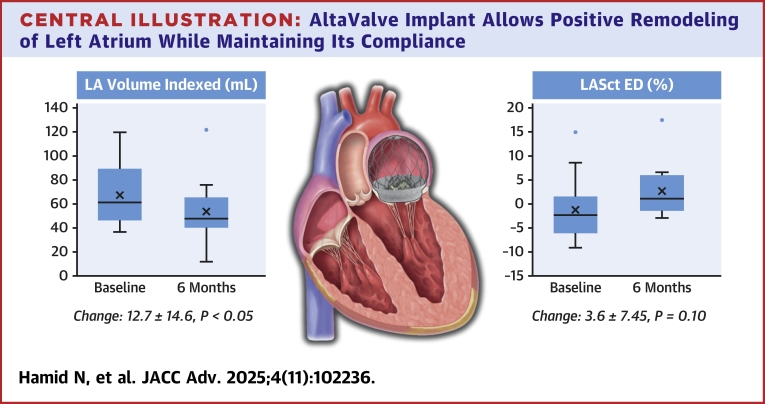
**AltaValve Implant Allows Positive Remodeling of Left Atrium While Maintaining Its Compliance** Atrial-fixation TMVR technology shows evidence of reverse-remodeling post implant, with reductions in LA volume indexed and LA contraction strain at end diastole (LASctED). LA = left atrium.

The study evaluated the impact of AltaValve System’s cage on LA function. Baseline LA dysfunction was attributed to severe MR and comorbidities, including atrial fibrillation. At 6 months, reduced reservoir strain reflected decreased LA compliance, seen in both groups. Impaired reservoir function limits preload adaptation, contributing to persistent heart failure symptoms. The AltaValve System TMVR showed no changes in the LA contractility, indicating minimal cage impact on the LA. Importantly, the device cage did not prevent favorable LA remodeling, which was seen in both responders and nonresponders suggesting a less risk of thrombus formation in the LA and along the LA wall and perhaps improved LA function, which can only be determined with longer-term follow-up.

Prior studies link reduced reservoir function to poorer outcomes postmitral surgery, regardless of baseline functional status.^2^ In our cohort, responders also showed greater reductions in LA volume and improved strain. Longer-term studies in larger populations are needed to assess sustained effects.

### Study Limitations

This was a retrospective analysis of the transthoracic echocardiography with a small sample size. Optimum echocardiographic views were required to avoid LA foreshortening and for accurate delineation of the LA endocardium. The images obtained after implantation of the AltaValve System TMVR can be challenging in some patients, due to the shadowing of the device.

## Conclusions

We find that there was early reduction in LA volumes and LA strain for both the reservoir and conduit phases without changes in contractility in patients who underwent AltaValve System TMVR implantation. These findings suggest favorable remodeling despite the “imprint” of the valve frame on the LA and its compliance with improvement in clinical outcomes. Further long-term analysis is warranted to evaluate the clinical impact of these findings.

Perspectives **COMPETENCY IN PATIENT CARE AND PROCEDURAL SKILLS:**

Patients who had AltaValve TMVR saw early reduction in LA volumes with changes in LA strain that does not impact LA contractility. These LA remodeling favor compliance and clinical improvements of these patients.

**TRANSLATIONAL OUTLOOK:**

Even in patients with significant mitral regurgitation and not deemed suitable for surgery, an alternative is a TMVR option. This approach may be considered a treatment option and shows not only elimination of mitral regurgitation but also left atrial remodeling and improvement in quality of life outcomes.

## Funding support and author disclosures

Dr Hamid has received consulting fees from Abbott Structural, 4C Medical, Philips Healthcare, GE, Siemens, Edwards Lifesciences, Vdyne and Laza. Dr Sorajja has received consulting fees from consulting for 4C Medical, Abbott Structural, Adona, Boston Scientific, CroiValve, ConKay, Cultiv8, Edwards Lifesciences, Egg Medical, Evolution Medical, Foldax, GE Healthcare, inQ8, Haemonetics, Laza, Medtronic, Philips, Polares, Unorthodox Ventures, ValCare, and vDyne. Drs Ninios and Wrobel have received speaker honoraria and compensation for proctoring from 4C Medical Technologies, Inc outside the submitted work. Dr Genereux has received speaker honoraria and consulting fees from 4C Medical Technologies, Inc outside the submitted work. Dr Genereux has received consulting fees from Edwards Lifesciences, Pi cardia, Medtronic, Haemonetics and Puzzle Medical. Dr Tahirkheli is an investor in 4C Medical Technologies, Inc. All other authors have reported that they have no relationships relevant to the contents of this paper to disclose.

## References

[1] Nishimura R.A., Otto C.M., Bonow R.O. (2017). 2017 AHA/ACC focused update of the 2014 AHA/ACC guideline for the management of patients with valvular heart disease: a report of the American College of Cardiology/American Heart Association task force on clinical practice guidelines. J Am Coll Cardiol.

[2] Shafii A.E., Gillinov A.M., Mihaljevic T., Stewart W., Batizy L.H., Blackstone E.H. (2012). Changes in left ventricular morphology and function after mitral valve surgery. Am J Cardiol.

